# P02.175. A randomized controlled pilot study assessing quality of life, stress and feasibility of yoga practice in women newly diagnosed with breast cancer

**DOI:** 10.1186/1472-6882-12-S1-P231

**Published:** 2012-06-12

**Authors:** S Pruthi, L Solberg Nes, J Boughey, M Huebner, B Borg, S Jenkins, D Stan, R Singh, S Kohli, V Lemaine

**Affiliations:** 1 Mayo Clinic, Rochester, USA

## Purpose

This study’s aim was to assess feasibility and the impact of yoga in improving quality of life for women after breast cancer diagnosis.

## Methods

With IRB approval a prospective randomized study was conducted of women newly diagnosed with breast cancer. Patients were randomized to yoga practice or control group. Both groups (15 patients in each) completed questionnaires and provided saliva samples at baseline and at 12 weeks for cortisol level evaluation. After surgery, the yoga group received 2 individual weekly sessions followed by 8 weekly group sessions and were provided with a gentle yoga DVD for home use at least once per week.

## Results

Lumpectomy rates were 67% in the yoga group and 47% in the control group. Among yoga participants, 13 attended the individual sessions, 11 the group classes (median of 3 classes), and 12 used the DVD at least weekly. Both groups had significant improvements in QOL scores from pre- to post-treatment: the median FACT-B emotional well-being score increased by 2.9 (yoga) and 3.7 (control); POMS tension-anxiety, depression-dejection, and confusion-bewilderment scores also improved (medians ranged from 0.5 to 0.9). There was no significant difference between the groups in level of improvement. Pre to post treatment median cortisol and cortisone levels also decreased; in the yoga group by 10 ng/dL and 86 ng/dL, respectively, and in the control group by 12 ng/dL and 74.5 ng/dL respectively. The yoga practice was rated as “very effective” for providing relaxation (85%), stress relief (69%), reduction of muscle tension (62%), and a general feeling of wellness (62%).

## Conclusion

From initial breast cancer diagnosis to post treatment there was significant improvement in emotional well-being, anxiety, depression and confusion. Cortisol levels decreased over this time period. In this small pilot study, addition of yoga did not produce a significant improvement in these variables when compared to the control group.

